# Supplementary material for: Acceptance and compliance with micronutrient powder (MNP) among children aged 6–23 months in northern Nigeria
Source: PLOS Glob Public Health. 2022 Oct 17;2(10):e0000961. doi: 10.1371/journal.pgph.0000961 (PMC10022258; doi:10.1371/journal.pgph.0000961)
Supplement: S1 Dataset — (ZIP) [file pgph.0000961.s008.zip › S8_Dataset/Direct Observations.zip!/Direct Observations/180615_A_SO_AS_1_DO.pdf]

1.1.1 Instrument F –Direct Observation Form

**<<Instrument F Direct Observation Form>>**
**PI Name:** PragyaMathema,Stephen Kodish
**Study Title:** Formative research to develop an infant and young child feeding (IYCF) intervention with micronutrient powder (MNP) for children aged 6 – 23 months
**PI Version/Date:** Version1.0/January 24, 2015

Date: ___18TH JUNE, 2015_____________Location: __SONG L.G.A, ADAMAWA____________________

Data Collector: __ANTIYA SANDA______________Participant ID: _A HUT BY THE ROAD SIDE__________

Main goals of this direct observation are to answer the following questions:

1. In this household, what would be the best way(s) to give nutritional supplements to young children? Why?

2. In this household, describe the food sharing that you observe throughout the meal. Are there any predictable patterns?

3. In this household, who is favored and how is this favoritism expressed during meal preparation and eating?

(TEAR OFF AT PERFORATED DOTTED LINE BELOW)

PARTICIPANT ID: _________

Household Composition Table (Fill out as you do data collection)

INITIALS	SEX (M/F)	AGE (estimated)	Relationship to household	Description
PB	M	35 YEARS	HUSBAND	A TALL DARK MAN WITH A PURPLE SHIRT
FB	F	32 YEARS	WIFE	A FAT BLACK WOMAN
SB	M	20 MONTHS	CHILD	A SMALL BLACK BOY
FC	M	4 YEARS	CHILD	A LITTLE CHUBBY BABY
SY	F	8 YEARS	CHILD	A SLIM GIRL WITH A YELLOW UNIFORM
TY	Y	6 YEARS	CHILD	A TALL YELLOW GIRL

SF	F	30 YEARS	NEIGHBOUR	A SLIM FAIR WOMAN

PARTICIPANT ID: __________

TIME	ACTOR INITIALS	RECIPIENT INITIALS	WHAT HAPPENED	FOOD	Reactivity
8:00	FB		SWEEPING THE HOUSE		
8:05	FB		SWEEPING THE HOUSE		
8:10	FB		SWEEPING THE HOUSE		
8:15	FB		WASHING THE PLATES		
8:20	FB		WASHING THE PLATES		
8:25	PB		WASHING HIS HANDS		
8:30	FB	FC	BREAST FEEDING		
8:40	FB	FC	BREAST FEEDING		
8:45	SB		LYING ON A MAT		
8:50	FB		BROUGHT FIRE WOOD		

TIME	ACTOR INITIALS	RECIPIENT INITIALS	WHAT HAPPENED	FOOD	Reactivity
9:00	FB		BLOWING THE FIRE WOOD		
9:05	FB		BLOWING THE FIRE WOOD		
9:10	FB		PUT THE POT ON FIRE		
9:11	FB		POUR WATER INTO THE POT ON FIRE		
9:12	FB		COVERS THE POT WITH THE COVER		
9:13	FB		CHECKED IF THE FIRE IS GLOWING		
9:14	FB		BROUGHT GUINEA CORN FLOUR	GUINEA CORN	
9:15	FB		MIXED WATER WITH GUINEA CORN FLOUR	GUINEA CORN	
9:16	FB		POUR THE PASTE INTO BOILING WATER	GUINEA CORN	
9:17	FB		STIR HOT WATER AND GUINEA CORN	GUINEA CORN	
9:18	FB		BROUGHT DOWN THE POT FROM THE FIRE	GUINEA CORN	
9:19	FB		POUR SOME KUNU PAP INTO A FLASK	GUINEA CORN	
9:20	FB		SERVES KUNU IN A SMALL CUP	GUINEA CORN	
9:21	FB		GIVES 2 CUPS OF KUNU TO BOTH CHILDREN	GUINEA CORN	
9:22	SB	SB	FEEDING HIMSELF WITH A CUP OF KUNU	GUINEA CORN	
9:23	SB	SB	STILL DRINKING HIS KUNU	GUINEA CORN	
9:24	SB	SB	FEEDING ON THE KUNU	GUINEA CORN	
9:25	SB	SB	FEEDING AND PLAYING	GUINEA CORN	
9:26	SB	SB	FEEDING AND PLAYING	GUINEA CORN	
9:27	SB	SB	FINISHES ALL HIS KUNU	GUINEA CORN	
9:28	SB		LYING ON THE MAT		

TIME	ACTOR INITIALS	RECIPIENT INITIALS	WHAT HAPPENED	FOOD	Reactivity
9:29	SG	SG	DRINKING HER KUNU		
9:30	SB		STARTS SLEEPING		
9:35	SB		SLEEPING		
9:40	SB		SLEEPING		
9:45	SB		SLEEPING		
9:50	SB		SLEEPING		
9:55	SB		SLEEPING		
10:00	SB		SLEEPING		
10:05	SB		SLEEPING		
10:10	SB		SLEEPING		
10:15	SB		SLEEPING		
10:20	SB		SLEEPING		
10:25	SB		SLEEPING		
10:30	SB		SLEEPING		
10:35	SB		SLEEPING		
10:40	SB		SLEEPING		
10:45	SB		SLEEPING		
10:50	SB		WAKES UP AND SAT DOWN		
10:55	SB		TOOK A CUP AND FETCH WATER FROM THE POT		
11:00	SB	SB	DRINKING WATER	WATER	

TIME	ACTOR INITIALS	RECIPIENT INITIALS	WHAT HAPPENED	FOOD	Reactivity
11:05	SB		GOES TO TOILET TO POOP		
11:10	SB		STILL IN THE TOILET		
11:15	SG	SB	WASHES HIS ENTIRE BODY		
11:20	SG	SB	STILL WASHING HIS BODY		
11:25	SB		STARTS PLAYING		
11:30	SB		PLAYING WITH A TOY		
11:35	SB		PLAYING		
11:40	SB		PLAYING		
11:45	SB		TAKES A CUP TO FETCH WATER		
11:50	SB	SB	DRINKING WATER	WATER	
11:55	SB		PLAYING		
12:00	SB		PLAYING		
12:05	SB		SITTING AND PLAYING		
12:10	SB		SITTING AND PLAYING		
12:15	SB		PLAYING IN THE COMPOUND		
12:20	SB		PLAYING IN THE COMPOUND		
12:25	SG	SB	FEEDING HIM WITH POWDER MILK	MILK POWDER	
12:30	SG	SB	FEEDING HIM WITH POWDERED MILK	MILK POWDER	
12:35	SB		TAKES A CUP TO FETCH WATER		
12:40	SB	SB	DRINKING WATER FROM A CUP	WATER	
12:45	TY	SB	BATHING HER BROTHER		

TIME	ACTOR INITIALS	RECIPIENT INITIALS	WHAT HAPPENED	FOOD	Reactivity
12:50	TY	SB	CLEANING HIS BODY WITH TOWEL		
12:55	SB		STARTS PLAYING		
1:00	SB		PLAYING		
1:05	SB		STILL PLAYING		
1:10	SB		PLAYING IN THE COMPOUND		
1:15	SF		BRINGS A BOWL WITH FOOD INSIDE		
1:20	TY	SB	FEEDING HIM WITH TUWO	MAIZE FLOUR	
1:25	TY	SB	EATING AND FEEDING HER BROTHER	MAIZE FLOUR AND BOUBAB SOUP	
1:30	SB	SB	FEEDING HIMSELF WITH TUWO	MAIZE FLOUR AND BOUBAB SOUP	
1:35	SB	SB	DRINKING WATER	WATER	
1:40	SB	SB	EATING THE TUWO ALONE	MAIZE FLOUR AND BOUBAB SOUP	
1:45	SB	SB	STILL HAND FEEDING HIMSELF	MAIZE FLOUR AND BOUBAB SOUP	
1:50	SB		GOES TO THE TOILET TO URINATE		
1:55	PB		FETCHING WATER TO POUR INTO A BIG POT		

TIME	ACTOR INITIALS	RECIPIENT INITIALS	WHAT HAPPENED	FOOD	Reactivity
2:00	PB	SB	WASHING THE HANDS OF HIS SON		
2:05	PB	SB	BATHING HIS SON		
2:10	PB	SB	CONTINUED TO BATH HIM		
2:15	FB		WASHING ALL THE POTS		
2:20	FB		SWEEPING THE HOUSE		
2:30	FB		SWEEPING		
2:35	SB		LYING ON A MAT		
2:40	SB		STARTS TO SLEEP		
2:45	SB		SLEEPING		
2:50	SB		SLEEPING		
2:55	SB		SLEEPING		
3:00	SB		SLEEPING		
3:05	TY	SB	COVERS HER BROTHER WITH A WRAPPER		
3:10	SB		SLEEPING		
3:15	FB		POUNDING PEPPER	PEPPER	
3:16	FB		POUNDING PEPPER	PEPPER	
3:17	FB		PEELING ONIONS	ONION	
3:18	FB		PEELING ONIONS	ONION	
3:19	FB		MAKES FIRE		
3:20	FB		BLOWING THE FIRE		

3:21	FB		PUTS A POT ON THE FIRE		
3:22	FB		POUR GROUND NUT OIL INTO POT	GROUNDNUT OIL	
3:23	FB		POURS ONIONS INTO HOT OIL	ONIONS AND GROUNDNUT OIL	
3:24	FB		POURS PEPPER INTO POT	PEPPER	
3:25	FB		STIRS BOTH ONION AND PEPPER	ONIONS AND PEPPER	
3:26	FB		STILL FRYING THE ONIONS AND PEPPER	ONIONS AND PEPPER	
3:27	FB		BROUGHT THE POT DOWN FROM FIRE		
3:28	FB		COVERS THE POT		
3:29	FB		BRINGS MAIZE FLOUR	MAIZE FLOUR	
3:30	FB		STARTS TO SIEVE THE MAIZE FLOUR	MAIZE FLOUR	
3:31	FB		SIEVING	MAIZE FLOUR	
3:32	FB		SIEVING	MAIZE FLOUR	
3:33	FB		SIEVING	MAIZE FLOUR	
3:34	FB		SIEVEING	MAIZE FLOUR	
3:35	FB		COVERED THE SIEVED FLOUR	MAIZE FLOUR	
3:40	FB		SITTING ON THE MAT		
3:45	SB		WAKES FROM SLEEP		
3:50	SB		FATCHES WATER TO DRINK		
3:55	SB		DRINKING WATER	WATER	
4:00	FB, PB		BOTH FETCHING WATER FROM THE WELL		
4:05	SB		PLAYING WITH HIS TOY		
4:10	SB		PLAYING		

4:15	SB, TY		PLAYING TOGETHER		
4:20	SB, TY		STILL PLAYING		
4:25	SB, TY		PLAYING		
4:30	SB, TY		PLAYING		
4:35	FB, SB		STILL ON THE SAME MAT		
4:40	FB	FC	BREAST FEEDING HIM		
4:50	FB	FC	BREAST FEEDING		
4:55	FB	FC	BREAST FEEDING		
5:00	SB		TAKES A CUP TO DRINK WATER		
5:10	SB	SB	DRINKING WATER	WATER	
5:15	SB		RUNNING ROUND THE COMPOUND		
5:20	SB		PLAYING AROUND		
5:30	SB		PLAYING AROUND		
5:35	SY, TY		FETCHUNG WATER FROM THE POT		
5:40	SY, TY		WASHING THE PLATE TOGETHER		
5:45	SB		SITTING ON A MAT		
5;50	FB		REARRANGING THE FIRE WOOD		
5:55	SB, PB		SITTING TOGETHER ON A MAT		
6:00	FB		BATHING HER YOUNG CHILD		

PARTICIPANT ID: __________

Primary Questions (Complete after you complete data collection):

From what you have seen observing THIS HOUSEHOLD, please answer the following questions in detail:

Question	Write your <u>detailed</u> response here
1. In this household, what would be the best way(s) to give nutritional supplements such as an MNP to young children to ensure compliance?<br><br>Why?	
2. In this household, who is favored during meal preparation or feeding?<br><br>How is this favoritism expressed?	
3. How could we promote the MNP supplement for this family that would attract their attention?<br><br>Who would they listen to?	
4. In this household, what would be the best ways to	

promote the MNP but limit how much it would be shared?<br><br>Why?	
5. What other issues and concerns did you see with this family that are important for us to consider with regard to introduction of an MNP?<br><br>Why?	
6. Based on your experience observing this child today, do you believe reactivity negatively impacted your ability to see the food-related behaviors of the child?<br><br>Please explain your answer in detail.	
